# Six-minute walk test to assess muscular fatigability and mobility in children with congenital myasthenic syndrome: a pilot study

**DOI:** 10.1590/1806-9282.20250307

**Published:** 2026-05-01

**Authors:** Numan Bulut, Güllü Aydın Yağcıoğlu, Aykut Demir, İpek Alemdaroğlu-Gürbüz, Göknur Haliloğlu, Öznur Tunca

**Affiliations:** 1 Hacettepe University, Faculty of Physical Therapy and Rehabilitation – Ankara, Turkey.; 2 University of Health Sciences, Gulhane Faculty of Health Sciences, Department of Orthotics and Prosthetics – Ankara, Turkey.; 3 Hacettepe University, Faculty of Medicine, Department of Pediatrics, Division of Pediatric Neurology – Ankara, Turkey.

**Keywords:** Congenital myasthenic syndrome, Six-minute walk test, Functional performance, Muscle fatigue, Outcome assessment

## Abstract

**OBJECTIVE:**

The aims of this study were to determine whether the six-minute walk test predicts muscular fatigability in ambulant children with congenital myasthenic syndrome and to examine the relationship between the six-minute walk test and mobility tests.

**METHODS:**

Eight children (four girls and four boys) with congenital myasthenic syndrome (mean age: 11.75±3.54 years) were included. Five children had *COLQ* variants. The distance of each minute for the six-minute walk test was determined to evaluate fatigability. The mobility tests included the 10-m walk/run test, rising from the floor, and climbing up and down four stairs.

**RESULTS:**

The mean six-minute walk test distance was 463.38±132.59 m. There was no difference between the first and last minute, between the first and last two minutes, and between any of the minutes during the six-minute walk test (p>0.05). The mean times for performing the 10-m walk/run test, rising from the floor, and climbing up and down four stairs were 8.59±3.36, 6.38±4.49, 4.38±5.07, and 3.35±3.69 s, respectively. The strong correlations were determined between six-minute walk test and all the mobility tests, except for climbing down four stairs (p< 0.05, r_s_=-0.72 to -0.83).

**CONCLUSION:**

The six-minute walk test did not provide sufficient evidence to predict muscular fatigability. However, this preliminary study suggested that the six-minute walk test may be used by clinicians and researchers to assess mobility in congenital myasthenic syndrome.

## INTRODUCTION

Congenital myasthenic syndrome (CMS) is a rare genetic disorder that forms a subset of neuromuscular diseases in which genetic problems lead to the dysfunction of proteins that form the neuromuscular junction^1^. The prevalence of CMS is 2.5–12.5/1,000,000, corresponding to approximately 1/10 of myasthenia gravis^2^. CMS is categorized according to different criteria, including genetic inheritance and pre-synaptic, synaptic, or post-synaptic defects. Variants in the genes that disrupt the function of proteins involved in the structure, function, and repair of the neuromuscular junction, such as *CHAT*, *CHRNE*, *RAPSN*, *DOK7*, and *COLQ*, are known to cause CMS^1^.

The presence of muscular symptoms is a key feature of CMS. These symptoms include ptosis, ophthalmoparesis, and weakness of the face, bulbar, trunk, and limbs, as well as dyspnea, hypotonia, and decreased tendon reflexes^3^. Although symptoms vary depending on genetic variants, fatigue, which is defined as an insufficiency of energy or the presence of weakness or burnout in mental, physical, or both dimensions^4^, is one of the most prominent symptoms of CMS^5^.

The six-minute walk test (6MWT) was originally developed to assess exercise capacity^6^. Over the years, it has been used to determine various parameters such as functional capacity and muscle strength^7^. In addition to having normative values, the 6MWT is known to be a valid and reliable method in neurological diseases, including myasthenia gravis, and is sensitive to change^8,9,10^.

It has been suggested that the 6MWT may demonstrate muscular fatigability and be associated with functional performance in neuromuscular diseases^11,12,13^. Eichinger et al. reported an association between the 6MWT and functional performance parameters in individuals with facioscapulohumeral muscular dystrophy (FSHD)^11^. Another study involving ambulant individuals with spinal muscular atrophy (SMA) also showed a decrease of approximately 9.5 m in the last minute compared to the first minute of the 6MWT, suggesting that the 6MWT is sensitive to fatigue-related changes^12^. In *RYR1*-associated myopathy, walking speed in the second, third, and fourth minutes during the 6MWT is reported to be lower than in the first minute, indicating fatigue^13^. A recent study examined the relationship between the instrumented 6MWT and physical activity, demonstrating a weak correlation between decreased walking distance and increased sedentary activity^14^.

Although timed performance tests and the 6MWT are recommended in CMS^15^, these parameters are not routinely used in the CMS field. The hypotheses of our study were that: (a) the 6MWT predicts muscular fatigability in children with CMS, and (b) the 6MWT is associated with other timed performance tests that indicate functional performance in CMS. In this direction, the aims of this study were twofold: to determine whether the 6MWT indicates muscular fatigability in ambulant children with CMS and to examine its relationship with mobility tests.

## METHODS

### Design

This retrospective study was conducted on children with CMS who were admitted to the Pediatric Neuromuscular Diseases Unit at the Faculty of Physical Therapy and Rehabilitation and were also followed up by the Division of Pediatric Neurology outpatient clinic, Department of Pediatrics, at the Faculty of Medicine between February 2021 and January 2024. Ethical approval was obtained from the Health Sciences Research Ethics Committee (January 09, 2024, Decision ID: SBA 24/058). Data were obtained in accordance with the Declaration of Helsinki.

### Participants

Children aged 5–18 years with genetically confirmed CMS who could walk independently and climb up stairs (Grade 1–3 according to the Vignos Scale) were included in the study. The study involved children who performed all the outcome measures in full. Children with an additional chronic condition, a lower extremity injury or surgery within the last 6 months, and poor cooperation were excluded.

### Outcome measures

The demographic characteristics of children with CMS were recorded. In addition, descriptive information related to the disease, such as age of onset of symptoms, medications, and genetic results, was obtained. The following assessments were performed in random order, with adequate rest intervals given between each one.

### Functional level

The Vignos Scale was used to evaluate the functional level of the children. The Vignos Scale is a functional scale that classifies the lower extremity function into eight different grades. Grade 1 refers to independent walking and stair climbing, while Grade 8 states to bed dependency^16^. Within the scope of the scale, the children were asked to walk and climb up four standard stairs with a handrail and a height of 15 cm. They were scored as Grade 1 if they walked and climbed up four stairs without assistance, Grade 2 if they walked and climbed up four stairs with the aid of a railing, and Grade 3 if they walked and climbed up four stairs slowly with the aid of a railing (over 12 s for four standard stairs).

### Six-minute walk test

The 6MWT was performed by two physiotherapists in accordance with the American Thoracic Society (ATS) guidelines^6^. Traffic cones were placed at the beginning and end of a 25-m corridor, and the child was asked to walk as fast as possible for 6 min without running. One physiotherapist walked 1–2 m behind the child as a safety chaser, while the other physiotherapist was timer at the beginning of the corridor. The timer shared completed and remaining minutes information, and the children were encouraged with standardized phrases at intervals of about 15 s. The patient was informed that the test could be stopped if they expressed excessive fatigue, palpitations, tachycardia, or pain. They were also informed that the time would not be stopped in case of a fall and that the test could be continued if they wanted to get up and carry on. However, if they did not want to continue, the test would be terminated. For the 6MWT, the distance walked was recorded in m^17^. In addition, the distance walked in each minute was obtained. Before and after the 6MWT, heart rate (HR) and oxygen saturation (SpO_2_) were measured using a pulse oximeter (G-Life YK-820C, Yonker Medical, Xuzhou, Jiangsu, China).

### Mobility tests

The 10-m walk/run test, rising from the floor, climbing up and down four stairs were used to evaluate the mobility skills of children. For the 10-m walk/run test, the patient was asked to complete 10 m. For the rising from the floor, they were asked to move from the supine position to the standing position with their arms by their sides. The children were expected to climb up and down four stairs of 15 cm height with a standard handrail, and time was recorded separately for climbing up and down stairs^17^. The tests were asked to be completed as fast as possible, and the time to complete the tests was recorded in s.

### Statistical analyses

SPSS Version 26.0 was used for data analysis. A Kolmogorov-Smirnov test was performed to determine whether the data were parametric. The data were determined to be non-parametric. Numerical variables were expressed as mean±standard deviation/median (interquartile range) and nominal/ordinal variables as n (%). To determine whether the 6MWT predicts muscle fatigability, the Friedman test was used to evaluate whether there was a difference in the distance covered each minute. Hemodynamics were compared using the Wilcoxon signed-rank test. Spearman’s correlation coefficient (r_s_) was used to evaluate the relationship between the 6MWT and mobility variables. The correlation coefficients (r_s_) were categorized as weak (0.01–0.39); moderate (0.4–0.69); and strong (0.7–0.99)^18^. A p-value of p<0.05 was considered statistically significant.

## RESULTS

Eight children who met the inclusion criteria were identified. As all eight children underwent all assessment parameters, there was no missing data in the study. The mean age of the children was 11.75±3.54 years. Demographic and disease descriptive characteristics of each child are presented in Table 1. Five (62.5%) of the children had *COLQ* variants. Respiratory deficiency (n=3), ptosis (n=3), muscle weakness (n=1), and ophthalmoplegia (n=1) were the main findings. The treatment regime included monoor combination therapy including salbutamol, pyridostigmine, ephedrine, and 3,4-diaminopyridine.

**Table 1:** Demographic and descriptive characteristics of children with congenital myasthenic syndrome.

	Gender	Mutation type	Age (years)	BMI (kg/m^2^)	Symptom onset age (years)	Functional level^*^	Family history	Consanguinity
C1	F	*COLQ*	10	17.16	3	1	No	Yes
C2	M	*COLQ*	15	32.05	5	3	No	Yes
C3	F	*CHRNE*	13	16.63	4	1	No	No
C4	F	*CHAT*	10	17.33	2	1	No	Yes
C5	M	*COLQ*	15	15.19	0	1	Yes	Yes
C6	M	*COLQ*	9	14.26	0	1	No	Yes
C7	M	*COLQ*	16	16.85	0	1	Yes	Yes
C8	F	*CHRNE*	6	12.80	1	1	No	No

*According to Vignos Scale; C: Case; *CHAT*: choline acetyltransferase; *CHRNE*: acetylcholine receptor epsilon (∊) subunit; *COLQ*: collagen like tail subunit of asymmetric acetylcholinesterase; F: female; M: male; BMI: body mass index.

The distance of the 6MWT and the results of the mobility tests are shown in Table 2. No difference was found between the first and last minute, the first and last two minutes, and any minutes during the 6MWT (p>0.05). The median HR was 107.50 beats per minute (bpm) before the test and 133.50 bpm after the test (p=0.012). The median SpO_2_ for the pre- and posttest was 97.50 and 97.00%, respectively (p>0.05).

**Table 2:** The results of the six-minute walk test and mobility tests of children with congenital myasthenic syndrome.

Variables	Children with congenital myasthenic syndrome (n=8)	
	Mean±SD	Median (IQR)
Walking distance at 1st min (m)	76.38±19.60	81.00 (56.25–89.25)
Walking distance at 2nd min (m)	76.25±19.70	78.50 (60.25–90.25)
Walking distance at 3rd min (m)	79.56±23.17	83.00 (61.25–98.00)
Walking distance at 4th min (m)	77.56±23.55	79.00 (60.25–97.75)
Walking distance at 5th min (m)	77.63±24.85	86.00 (56.50–94.00)
Walking distance at 6th min (m)	75.75±24.99	83.00 (52.50–92.50)
Six-minute walk test (m)	463.38±132.59	490.50 (354.50–558.00)
10-m walk/run test (s)	8.59±3.36	7.67 (5.93–11.31)
Rising from the floor (s)	6.38±4.49	5.14 (3.31–7.94)
Climbing up four stairs (s)	4.38±5.07	2.55 (2.30–3.60)
Climbing down four stairs (s)	3.35±3.69	2.22 (1.74–2.54)

IQR: interquartile range; SD: standard deviation.

Table 3 shows the correlations between the evaluation parameters of the children. The 6MWT distance was found to be negatively correlated with the mobility tests except for climbing down four stairs (p<0.05, r_s_=-0.72 to -0.83). Similarly, the distances covered each minute were found to be correlated with each other (p<0.05).

**Table 3:** The relation between the six-minute walk test and mobility tests in children with congenital myasthenic syndrome.

Measures		Six-minute walk test	10-m walk/run test	Rising from the floor	Climbing up four stairs	Climbing down four stairs
Six-minute walk test	r_s_		-0.81	-0.83	-0.72	-0.21
	p		0.02^*^	0.01^*^	0.04^*^	0.61
10-m walk/run test	r_s_	-0.81		0.74	0.44	-0.10
	p	0.02^*^		0.04^*^	0.28	0.82
Rising from the floor	r_s_	-0.83	0.74		0.68	0.50
	p	0.01^*^	0.04^*^		0.06	0.21
Climbing up four stairs	r_s_	-0.72	0.44	0.68		0.64
	p	0.04^*^	0.28	0.06		0.09
Climbing down four stairs	r_s_	-0.21	-0.10	0.50	0.64	
	p	0.61	0.82	0.21	0.09	

r_s_: Spearman’s correlation coefficients

*p<0.05.

## DISCUSSION

This retrospective study was conducted to determine whether the 6MWT is an indicator of fatigability and whether it is associated with mobility in children with CMS. Within this small cohort of ambulant pediatric CMS patients, the 6MWT did not provide additional insight into fatigability. However, the exploratory findings suggest that it could be useful for assessing mobility.

In a CMS cohort of 69 patients, 58% were reported to have *CHRNE* variants, with 75% having onset of symptoms in infancy, and *CHRNE* variants were followed by *COLQ* variants presenting with severe myasthenic symptoms, including bulbar involvement, ptosis, and respiratory insufficiency^19^. Gül Mert et al. reported that five out of eight patients with the *COLQ* variant were able to walk independently and climb up stairs without support by the end of treatment^20^. Despite conflicting findings in the literature, the majority (62.5%) of patients in the current study had a *COLQ* variant. However, factors such as age of onset, symptom severity, and functional status may be influenced by various parameters such as variant site and treatment received.

The 6MWT and mobility tests, such as rising from the floor and climbing up the stairs, are important outcome measures used in individuals who can walk to show treatment efficacy and to monitor disease prognosis in many chronic pathologies^16,21^. The use of 6MWT and mobility tests in CMS is also recommended in a recent workshop^15^. Compared with the norm values of typically developing peers^8,22^, children with CMS were found to walk approximately 60 m less during a six-minute walk test and take around four times longer to rise from the floor, indicating poor performance in all assessment parameters. These results may show that the suggestions given in the current workshop^15^ are proper. They may also provide the first evidence that they can be used to assess normal-pathological differentiation and disease follow-up.

Although fatigue is one of the main motor symptoms in myasthenic syndromes and there are many ways to assess fatigue in adults, these methods are limited when it comes to congenital forms of the condition. In our study, we aimed to address this gap in the literature. However, the 6MWT did not provide sufficient evidence to demonstrate muscular fatigability in CMS. Opinions differ on whether the 6MWT can predict muscular fatigability in studies of various neuromuscular diseases, including SMA and *RYR1*-related myopathy^12,13^. However, there was no difference between the first two minutes and the last two minutes of the 6MWT in FSHD. It was also shown that, in children with DMD, the walking distance during each minute of the 6MWT remained stable and that the 6MWT did not provide any additional information regarding fatigability compared to the 2MWT^23^. Similarly, this study reported that more time did not provide any extra information in terms of fatigability. Although an increase in HR is an expected physiological response to activity such as walking, the stable SpO_2_ levels during the test support this view.

The 6MWT is known to be associated with many parameters such as physical activity, muscle strength, and balance in neurological diseases^7^. Different outcome measures to assess mobility were used in this study. The 6MWT showed correlations with all mobility parameters, except for climbing down four stairs. The study’s hypothesis was accepted, and presenting these correlations in the CMS, as well as in many neuromuscular diseases, suggests that the 6MWT can be used to assess mobility in these diseases. While there is a high level of correlation, given the small number of participants, it is necessary to confirm these findings with a larger sample size. However, the lack of correlation between climbing down four stairs and the 6MWT may be due to the limited number of participants. This discrepancy may be explained by the biomechanical nature of climbing down stairs, which involves factors such as fear of falling, increased joint peak power, negative work, eccentric contraction, and activation of different muscle groups compared to climbing up stairs and walking^24,25^.

This study has several limitations. Notably, the small sample size (n=8) restricts the study’s statistical power and generalizability. However, recruiting large cohorts is challenging because CMS is an orphan disease, and only children who can climb up stairs are included. The study was single-centered and retrospective, which may also be considered among the limitations. Although retrospective studies are prone to potential bias, the absence of missing data can be considered as one of the study’s strengths. Other limitations include the lack of a control group and a perceived fatigue scale, such as the Borg Scale. Future prospective controlled studies should include longitudinal designs with large cohorts, using different endurance assessments such as the sit-to-stand test and the trunk flexion test, and technological wearable devices (e.g., actigraphy), as well as assessing quality of life, muscle strength, nutrition, and respiratory parameters. There is a need for future multicenter collaborative studies to increase the sample size and improve the robustness of the evidence, as this will help to provide more reliable results. Qualitative feedback from participants could also be used in the future to explore physiological or behavioral factors affecting activity and mobility.

## CONCLUSION

To the best of our knowledge, this is the first study to use the 6MWT and mobility tests in a pediatric CMS cohort. The 6MWT did not provide sufficient evidence to predict muscular fatigability. However, this study presented preliminary findings suggesting that the 6MWT may be a suitable tool for assessing mobility.

## Data Availability

The datasets generated and/or analyzed during the current study are available from the corresponding author upon reasonable request.
